# Supplementary material for: Investigating Polarity Effects in DNA Base Stacking
Source: JACS Au. 2025 Nov 28;5(12):5924–31. doi: 10.1021/jacsau.5c01318 (PMC12728633; doi:10.1021/jacsau.5c01318)
Supplement: Supplementary file 1 [file au5c01318_si_001.pdf]

Supporting Information:

Investigating polarity effects in DNA base stacking

Jibin Abraham Punnoose, Chai Kam, Tristan Melfi, Sweta Vangaveti, Alan Chen, Ken Halvorsen.

Contents

Table S1: List of oligonucleotides

Table S2: Oligonucleotide combinations for each single-molecule construct

Table S3: Construct combination to form tethers with preferred base-stacking combination

Figure S1: Decay plot and single-exponential fitting of A|G, C|A, and C|G combinations of base-stacks at 15 pN

Figure S2: Decay plot and single-exponential fitting of T|A, T|G combinations and control construct at 15 pN

Figure S3: Decay plot and single-exponential fitting of T|C base-stack and corresponding control constructs at 15 pN

Figure S4: Polar plots for stacked bases in all MD simulations.

Figure S5: Polar plots for A|C and C|A stacking interactions with K+ ions at neutralizing or excess concentration.

Figure S6: Detailed molecular interface for C|G stacking

Table S1. List of oligonucleotide sequences.

Name	Sequence	Length
**Backbone sequences (5′-3′) (Common set of oligos for all single-molecule constructs)**		
1. 5'Biotin	(5' 2x bio) AACATCCAATAAATCATACAGGCAAGGCAAAGAATTAGCA	40
2	AAATTAAGCAATAAAGCCTC	20
3	AGAGCATAAAGCTAAATCGGTTGTACCAAAAACATTATGACCCTGTAATACTTTTGCGGG	60
4	AGAAGCCTTTATTTCAACGCAAGGATAAAAATTTTTAGAACCCTCATATATTTTAAATGC	60
5	AATGCCTGAGTAATGTGTAGGTAAAGATTCAAAAGGGTGAGAAAGGCCGGAGACAGTCAA	60
6	ATCACCATCAATATGATATTCAACCGTTCTAGCTGATAAATTAATGCCGGAGAGGGTAGC	60
7	TATTTTTGAGAGATCTACAAAGGCTATCAGGTCATTGCCTGAGAGTCTGGAGCAAACAAG	60
8	AGAATCGATGAACGGTAATCGTAAAACTAGCATGTCAATCATATGTACCCCGGTTGATAA	60
9	TCAGAAAAGCCCCAAAAACAGGAAGATTGTATAAGCAAATATTTAAATTGTAAACGTTAA	60
10	TATTTTGTTAAAATTCGCATTAAATTTTTGTTAAATCAGCTCATTTTTTAACCAATAGGA	60
11	ACGCCATCAAAAATAATTCGCGTCTGGCCTTCCTGTAGCCAGCTTTCATCAACATTAAAT	60
12	GTGAGCGAGTAACAACCCGTCGGATTCTCCGTGGGAACAAACGGCGGATTGACCGTAATG	60
13	GGATAGGTCACGTTGGTGTAGATGGGCGCATCGTAACCGTGCATCTGCCAGTTTGAGGGG	60
14	ACGACGACAGTATCGGCCTCAGGAAGATCGCACTCCAGCCAGCTTTCCGGCACCGCTTCT	60
15	GGTGCCGGAAACCAGGCAAAGCGCCATTCGCCATTCAGGCTGCGCAACTGTTGGGAAGGG	60
16	CGATCGGTGCGGGCCTCTTCGCTATTACGCCAGCTGGCGAAAGGGGGATGTGCTGCAAGG	60
17	CGATTAAGTTGGGTAACGCCAGGGTTTTCCCAGTCACGACGTTGTAAAACGACGGCCAGT	60
18	GCCAAGCTTGCATGCCTGCAGGTCGACTCTAGAGGATCCCCGGGTACCGAGCTCGAATTC	60
19	GTAATCATGGTCATAGCTGTTTCCTGTGTGAAATTGTTATCCGCTCACAATTCCACACAA	60
20	CATACGAGCCGGAAGCATAAAGTGTAAAGCCTGGGGTGCCTAATGAGTGAGCTAACTCAC	60
21	ATTAATTGCGTTGCGCTCACTGCCCGCTTTCCAGTCGGGAAACCTGTCGTGCCAGCTGCA	60
22	TTAATGAATCGGCCAACGCGCGGGGAGAGGCGGTTTGCGTATTGGGCGCCAGGGTGGTTT	60
23	TTCTTTTCACCAGTGAGACGGGCAACAGCTGATTGCCCTTCACCGCCTGGCCCTGAGAGA	60
24	GTTGCAGCAAGCGGTCCACGCTGGTTTGCCCCAGCAGGCGAAAATCCTGTTTGATGGTGG	60
25	TTCCGAAATCGGCAAAATCCCTTATAAATCAAAAGAATAGCCCGAGATAGGGTTGAGTGT	60
26	TGTTCCAGTTTGGAACAAGAGTCCACTATTAAAGAACGTGGACTCCAACGTCAAAGGGCG	60
27	AAAAACCGTCTATCAGGGCGATGGCCCACTACGTGAACCATCACCCAAATCAAGTTTTTT	60
28	GGGGTCGAGGTGCCGTAAAGCACTAAATCGGAACCCTAAAGGGAGCCCCCGATTTAGAGC	60
29	TTGACGGGGAAAGCCGGCGAACGTGGCGAGAAAGGAAGGGAAGAAAGCGAAAGGAGCGGG	60
30	CGCTAGGGCGCTGGCAAGTGTAGCGGTCACGCTGCGCGTAACCACCACACCCGCCGCGCT	60
31	TAATGCGCCGCTACAGGGCGCGTACTATGGTTGCTTTGACGAGCACGTATAACGTGCTTT	60
32	CCTCGTTAGAATCAGAGCGGGAGCTAAACAGGAGGCCGATTAAAGGGATTTTAGACAGGA	60
33	ACGGTACGCCAGAATCCTGAGAAGTGTTTTTATAATCAGTGAGGCCACCGAGTAAAAGAG	60
34	TCTGTCCATCACGCAAATTAACCGTTGTAGCAATACTTCTTTGATTAGTAATAACATCAC	60
35	TTGCCTGAGTAGAAGAACTCAAACTATCGGCCTTGCTGGTAATATCCAGAACAATATTAC	60

36	CGCCAGCCATTGCAACAGGAAAAACGCTCATGGAAATACCTACATTTTGACGCTCAATCG	60
37	TCTGAAATGGATTATTTACATTGGCAGATTCACCAGTCACACGACCAGTAATAAAAGGGA	60
38	CATTCTGGCCAACAGAGATAGAACCCTTCTGACCTGAAAGCGTAAGAATACGTGGCACAG	60
39	ACAATATTTTTGAATGGCTATTAGTCTTTAATGCGCGAACTGATAGCCCTAAAACATCGC	60
40	CATTAAAAATACCGAACGAACCACCAGCAGAAGATAAAACAGAGGTGAGGCGGTCAGTAT	60
41	TAACACCGCCTGCAACAGTGCCACGCTGAGAGCCAGCAGCAAATGAAAAATCTAAAGCAT	60
42	CACCTTGCTGAACCTCAAATATCAAACCCTCAATCAATATCTGGTCAGTTGGCAAATCAA	60
43	CAGTTGAAAGGAATTGAGGAAGGTTATCTAAAATATCTTTAGGAGCACTAACAACTAATA	60
44	GATTAGAGCCGTCAATAGATAATACATTTGAGGATTTAGAAGTATTAGACTTTACAAACA	60
45	ATTCGACAACTCGTATTAAATCCTTTGCCCGAACGTTATTAATTTTAAAAGTTTGAGTAA	60
46	CATTATCATTTTGCGGAACAAAGAAACCACCAGAAGGAGCGGAATTATCATCATATTCCT	60
47	GATTATCAGATGATGGCAATTCATCAATATAATCCTGATTGTTTGGATTATACTTCTGAA	60
48	TAATGGAAGGGTTAGAACCTACCATATCAAAATTATTTGCACGTAAAACAGAAATAAAGA	60
49	AATTGCGTAGATTTTCAGGTTTAACGTCAGATGAATATACAGTAACAGTACCTTTTACAT	60
50	CGGGAGAAACAATAACGGATTCGCCTGATTGCTTTGAATACCAAGTTACAAAATCGCGCA	60
51	GAGGCGAATTATTCATTTCAATTACCTGAGCAAAAGAAGATGATGAAACAAACATCAAGA	60
52	AAACAAAATTAATTACATTTAACAATTTCATTTGAATTACCTTTTTTAATGGAAACAGTA	60
53	CATAAATCAATATATGTGAGTGAATAACCTTGCTTCTGTAAATCGTCGCTATTAATTAAT	60
54	TTTCCCTTAGAATCCTTGAAAACATAGCGATAGCTTAGATTAAGACGCTGAGAAGAGTCA	60
55	ATAGTGAATTTATCAAAATCATAGGTCTGAGAGACTACCTTTTTAACCTCCGGCTTAGGT	60
56	TGGGTTATATAACTATATGTAAATGCTGATGCAAATCCAATCGCAAGACAAAGAACGCGA	60
57	GAAAACTTTTTCAAATATATTTTAGTTAATTTCATCTTCTGACCTAAATTTAATGGTTTG	60
58	AAATACCGACCGTGTGATAAATAAGGCGTTAAATAAGAATAAACACCGGAATCATAATTA	60
59	CTAGAAAAAGCCTGTTTAGTATCATATGCGTTATACAAATTCTTACCAGTATAAAGCCAA	60
60	CGCTCAACAGTAGGGCTTAATTGAGAATCGCCATATTTAACAACGCCAACATGTAATTTA	60
61	GGCAGAGGCATTTTCGAGCCAGTAATAAGAGAATATAAAGTACCGACAAAAGGTAAAGTA	60
62	ATTCTGTCCAGACGACGACAATAAACAACATGTTCAGCTAATGCAGAACGCGCCTGTTTA	60
63	TCAACAATAGATAAGTCCTGAACAAGAAAAATAATATCCCATCCTAATTTACGAGCATGT	60
64	AGAAACCAATCAATAATCGGCTGTCTTTCCTTATCATTCCAAGAACGGGTATTAAACCAA	60
65	GTACCGCACTCATCGAGAACAAGCAAGCCGTTTTTATTTTCATCGTAGGAATCATTACCG	60
66	CGCCCAATAGCAAGCAAATCAGATATAGAAGGCTTATCCGGTATTCTAAGAACGCGAGGC	60
67	GTTTTAGCGAACCTCCCGACTTGCGGGAGGTTTTGAAGCCTTAAATCAAGATTAGTTGCT	60
68	ATTTTGCACCCAGCTACAATTTTATCCTGAATCTTACCAACGCTAACGAGCGTCTTTCCA	60
69	GAGCCTAATTTGCCAGTTACAAAATAAACAGCCATATTATTTATCCCAATCCAAATAAGA	60
70	AACGATTTTTTGTTTAACGTCAAAAATGAAAATAGCAGCCTTTACAGAGAGAATAACATA	60
71	AAAACAGGGAAGCGCATTAGACGGGAGAATTAACTGAACACCCTGAACAAAGTCAGAGGG	60
72	TAATTGAGCGCTAATATCAGAGAGATAACCCACAAGAATTGAGTTAAGCCCAATAATAAG	60
73	AGCAAGAAACAATGAAATAGCAATAGCTATCTTACCGAAGCCCTTTTTAAGAAAAGTAAG	60
74	CAGATAGCCGAACAAAGTTACCAGAAGGAAACCGAGGAAACGCAATAATAACGGAATACC	60
75	CAAAAGAACTGGCATGATTAAGACTCCTTATTACGCAGTATGTTAGCAAACGTAGAAAAT	60
76	ACATACATAAAGGTGGCAACATATAAAAGAAACGCAAAGACACCACGGAATAAGTTTATT	60
77	TTGTCACAATCAATAGAAAATTCATATGGTTTACCAGCGCCAAAGACAAAAGGGCGACAT	60
78	TCAACCGATTGAGGGAGGGAAGGTAAATATTGACGGAAATTATTCATTAAAGGTGAATTA	60

79	TCACCGTCACCGACTTGAGCCATTTGGGAATTAGAGCCAGCAAAATCACCAGTAGCACCA	60
80	TTACCATTAGCAAGGCCGGAAACGTCACCAATGAAACCATCGATAGCAGCACCGTAATCA	60
81	GTAGCGACAGAATCAAGTTTGCCTTTAGCGTCAGACTGTAGCGCGTTTTCATCGGCATTT	60
82	TCGGTCATAGCCCCCTTATTAGCGTTTGCCATCTTTTCATAATCAAAATCACCGGAACCA	60
83	GAGCCACCACCGGAACCGCCTCCCTCAGAGCCGCCACCCTCAGAACCGCCACCCTCAGAG	60
84	CCACCACCCTCAGAGCCGCCACCAGAACCACCACCAGAGCCGCCGCCAGCATTGACAGGA	60
85	GGTTGAGGCAGGTCAGACGATTGGCCTTGATATTCACAAACAAATAAATCCTCATTAAAG	60
86	CCAGAATGGAAAGCGCAGTCTCTGAATTTACCGTTCCAGTAAGCGTCATACATGGCTTTT	60
87	GATGATACAGGAGTGTACTGGTAATAAGTTTTAACGGGGTCAGTGCCTTGAGTAACAGTG	60
88	CCCGTATAAACAGTTAATGCCCCCTGCCTATTTCGGAACCTATTATTCTGAAACATGAAA	60
89	GTATTAAGAGGCTGAGACTCCTCAAGAGAAGGATTAGGATTAGCGGGGTTTTGCTCAGTA	60
90	CCAGGCGGATAAGTGCCGTCGAGAGGGTTGATATAAGTATAGCCCGGAATAGGTGTATCA	60
91	CCGTACTCAGGAGGTTTAGTACCGCCACCCTCAGAACCGCCACCCTCAGAACCGCCACCC	60
92	TCAGAGCCACCACCCTCATTTTCAGGGATAGCAAGCCCAATAGGAACCCATGTACCGTAA	60
93	CACTGAGTTTCGTCACCAGTACAAACTACAACGCCTGTAGCATTCCACAGACAGCCCTCA	60
94	TAGTTAGCGTAACGATCTAAAGTTTTGTCGTCTTTCCAGACGTTAGTAAATGAATTTTCT	60
95	GTATGGGATTTTGCTAAACAACTTTCAACAGTTTCAGCGGAGTGAGAATAGAAAGGAACA	60
96	ACTAAAGGAATTGCGAATAATAATTTTTTCACGTTGAAAATCTCCAAAAAAAAAGGCTCCA	60
97	AAAGGAGCCTTTAATTGTATCGGTTTATCAGCTTGCTTTCGAGGTGAATTTCTTAAACAG	60
98	CTTGATACCGATAGTTGCGCCGACAATGACAACAACCATCGCCCACGCATAACCGATATA	60
99	TTCGGTCGCTGAGGCTTGCAGGGAGTTAAAGGCCGCTTTTGCGGGATCGTCACCCTCAGC	60
100	AGCGAAAGACAGCATCGGAACGAGGGTAGCAACGGCTACAGAGGCTTTGAGGACTAAAGA	60
101	CTTTTTCATGAGGAAGTTTCCATTAAACGGGTAAAATACGTAATGCCACTACGAAGGCAC	60
102	CAACCTAAAACGAAAGAGGCAAAAGAATACACTAAAACACTCATCTTTGACCCCCAGCGA	60
103	TTATACCAAGCGCGAAACAAAGTACAACGGAGATTTGTATCATCGCCTGATAAATTGTGT	60
104	CGAAATCCGCGACCTGCTCCATGTTACTTAGCCGGAACGAGGCGCAGACGGTCAATCATA	60
105	AGGGAACCGAACTGACCAACTTTGAAAGAGGACAGATGAACGGTGTACAGACCAGGCGCA	60
106	TAGGCTGGCTGACCTTCATCAAGAGTAATCTTGACAAGAACCGGATATTCATTACCCAAA	60
107	TCAACGTAACAAAGCTGCTCATTCAGTGAATAAGGCTTGCCCTGACGAGAAACACCAGAA	60
108	CGAGTAGTAAATTGGGCTTGAGATGGTTTAATTTCAACTTTAATCATTGTGAATTACCTT	60
109	ATGCGATTTTAAGAACTGGCTCATTATACCAGTCAGGACGTTGGGAAGAAAAATCTACGT	60
110	TAATAAAACGAACTAACGGAACAACATTATTACAGGTAGAAAGATTCATCAGTTGAGATT	60
111	TAGGAATACCACATTCAACTAATGCAGATACATAACGCCAAAAGGAATTACGAGGCATAG	60
112	TAAGAGCAACACTATCATAACCCTCGTTTACCAGACGACGATAAAAACCAAAATAGCGAG	60
113	AGGCTTTTGCAAAAGAAGTTTTGCCAGAGGGGGTAATAGTAAAATGTTTAGACTGGATAG	60
114	CGTCCAATACTGCGGAATCGTCATAAATATTCATTGAATCCCCCTCAAATGCTTTAAACA	60
115	GTTCAGAAAACGAGAATGACCATAAATCAAAAATCAGGTCTTTACCCTGACTATTATAGT	60
116	CAGAAGCAAAGCGGATTGCATCAAAAAGATTAAGAGGAAGCCCGAAAGACTTCAAATATC	60
117	GCGTTTTAATTCGAGCTTCAAAGCGAACCAGACCGGAAGCAAACTCCAACAGGTCAGGAT	60
118	TAGAGAGTACCTTTAATTGCTCCTTTTGATAAGAGGTCATTTTTGCGGATGGCTTAGAGC	60
119	TTAATTGCTGAATATAATGCTGTAGCTCAACATGTTTTAAATATGCAACTAAAGTACGGT	60
120	GTCTGGAAGTTTCATTCCATATAACAGTTGATTCCCAATTCTGCGAACGAGTAGATTTAG	60
121	TTTGACCATTAGATACATTTCGCAAATGGTCAATAACCTGTTTAGCTAT	49

122	ATTTTCATTTGGGGCGCGAGCTGAAAAGGT	30
CutOligo	CTACTAATAGTAGTAGCATTAACATCCAATAAATCATACA	40
**Sequence used in specific combination for each construct (5′-3′)**<br>**Overhanging regions underlined, spacer T are marked in blue, and stacking bases in red**		
OH-A	GGCATCAATTCTACTAATAGTAGTAGCATTCCGTGCCTGTGAACGAGCTGCCCCATGGCA	60
OH-G	GGCATCAATTCTACTAATAGTAGTAGCATTCCGTGCCTGTGAACGAGCTGCCCCATGGCG	60
OH-C	GGCATCAATTCTACTAATAGTAGTAGCATTCCGTGCCTGTGAACGAGCTGCCCCATGGCC	60
OH-T	GGCATCAATTCTACTAATAGTAGTAGCATTCCGTGCCTGTGAACGAGCTGCCCCATGGCT	60
A:C-A	ACGTCGCCTGCCATGGGGCAGCTCGTTCACAGGCACGG	38
T:T-G	GGCGACGTAGCCATGGGGCAGCTCGTTCACAGGCACGG	38
T:C-A	ACGTCGCCAGCCATGGGGCAGCTCGTTCACAGGCACGG	38
C:T-G	GGCGACGTGGCCATGGGGCAGCTCGTTCACAGGCACGG	38
C:C-A	ACGTCGCCGGCCATGGGGCAGCTCGTTCACAGGCACGG	38
T:G-T	TGCAGCGGAGCCATGGGGCAGCTCGTTCACAGGCACGG	38
T: Sp-T-G	GGCGACGTTTTAGCCATGGGGCAGCTCGTTCACAGGCACGG	41
T: Sp-C-A	ACGTCGCCTTTAGCCATGGGGCAGCTCGTTCACAGGCACGG	41
T: Sp-A-C	CCGCTGCATTTAGCCATGGGGCAGCTCGTTCACAGGCACGG	41
T: Sp-G-T	TGCAGCGGTTTAGCCATGGGGCAGCTCGTTCACAGGCACGG	41

Table S2: Oligonucleotide combinations for each single-molecule construct.

Construct #	Oligo Mix (1:15:50 mole ratio)
1	Oligos 1-122, OH-A, A:C-A
2	Oligos 1-122, OH-T, T:T-G
3	Oligos 1-122, OH-T, T:C-A
4	Oligos 1-122, OH-C, C:T-G
5	Oligos 1-122, OH-C, C:C-A
6	Oligos 1-122, OH-T, T:G-T
7	Oligos 1-122, OH-T, T:Sp-T-G
8	Oligos 1-122, OH-T, T:Sp-C-A
9	Oligos 1-122, OH-T, T:Sp-A-C
10	Oligos 1-122, OH-T, T:Sp-G-T

Table S3: Construct combination to form tethers in single-molecule experiments.

Tether ID	Stacking Combination (5′|3′)	Construct combinations to form tether
1	A|G	1& 7
2	T|A	2 & 8
3	T|G	3 & 7
4	C|A	4 & 8
5	C|G	5 & 7
8	Control for (A|G, T|A, T|G, C|A, C|G)	7 & 8

9	T|C	6 & 9
12	Control for (T|C)	9 &10

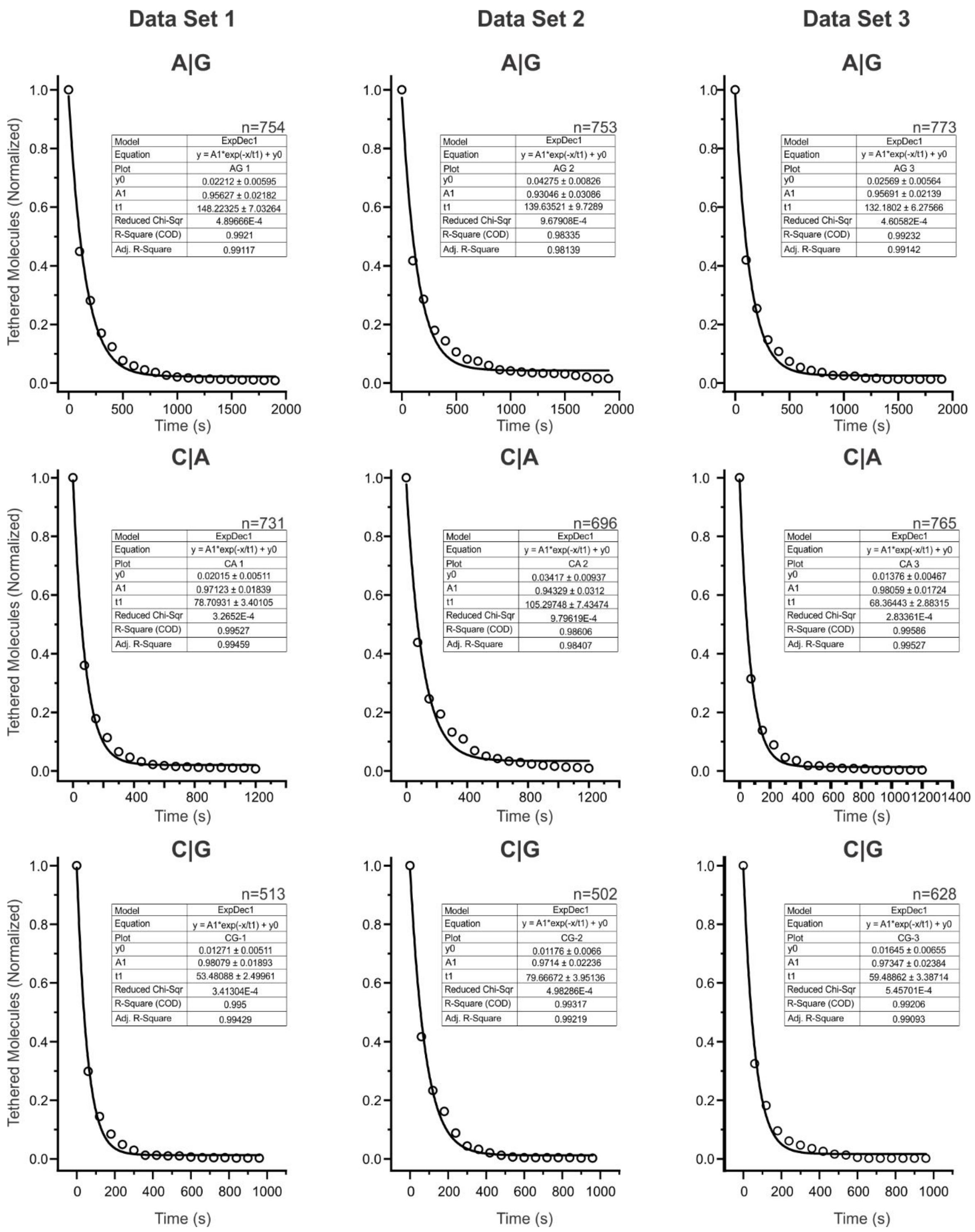

Figure S1: Decay plot and single-exponential fitting of A|G, C|A, and C|G combinations of base-stacks at 15 pN.

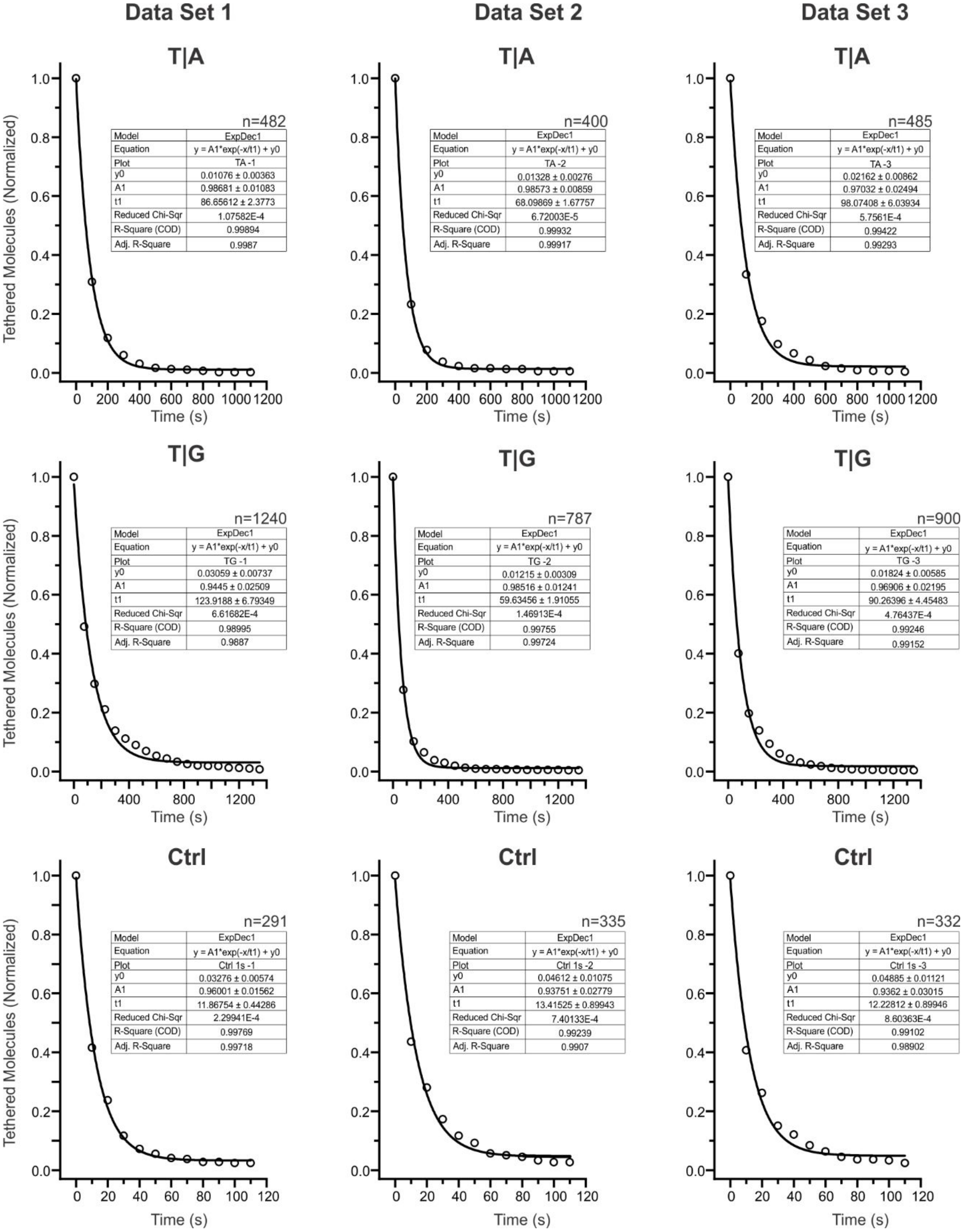

Figure S2: Decay plot and single-exponential fitting of T|A, T|G combinations and control construct at 15 pN.

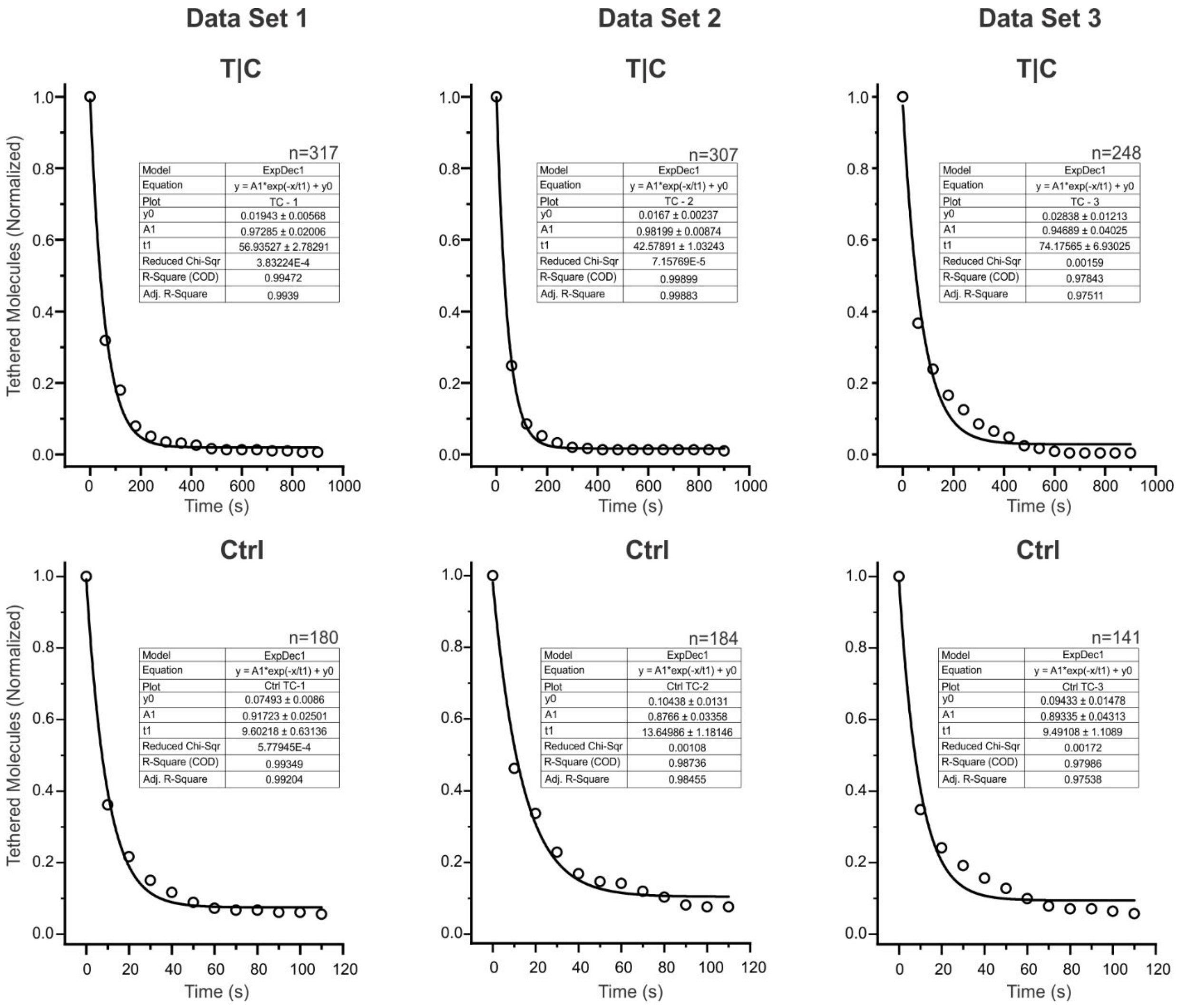

Figure S3: Decay plot and single-exponential fitting of T|C base-stack and corresponding control constructs at 15 pN.

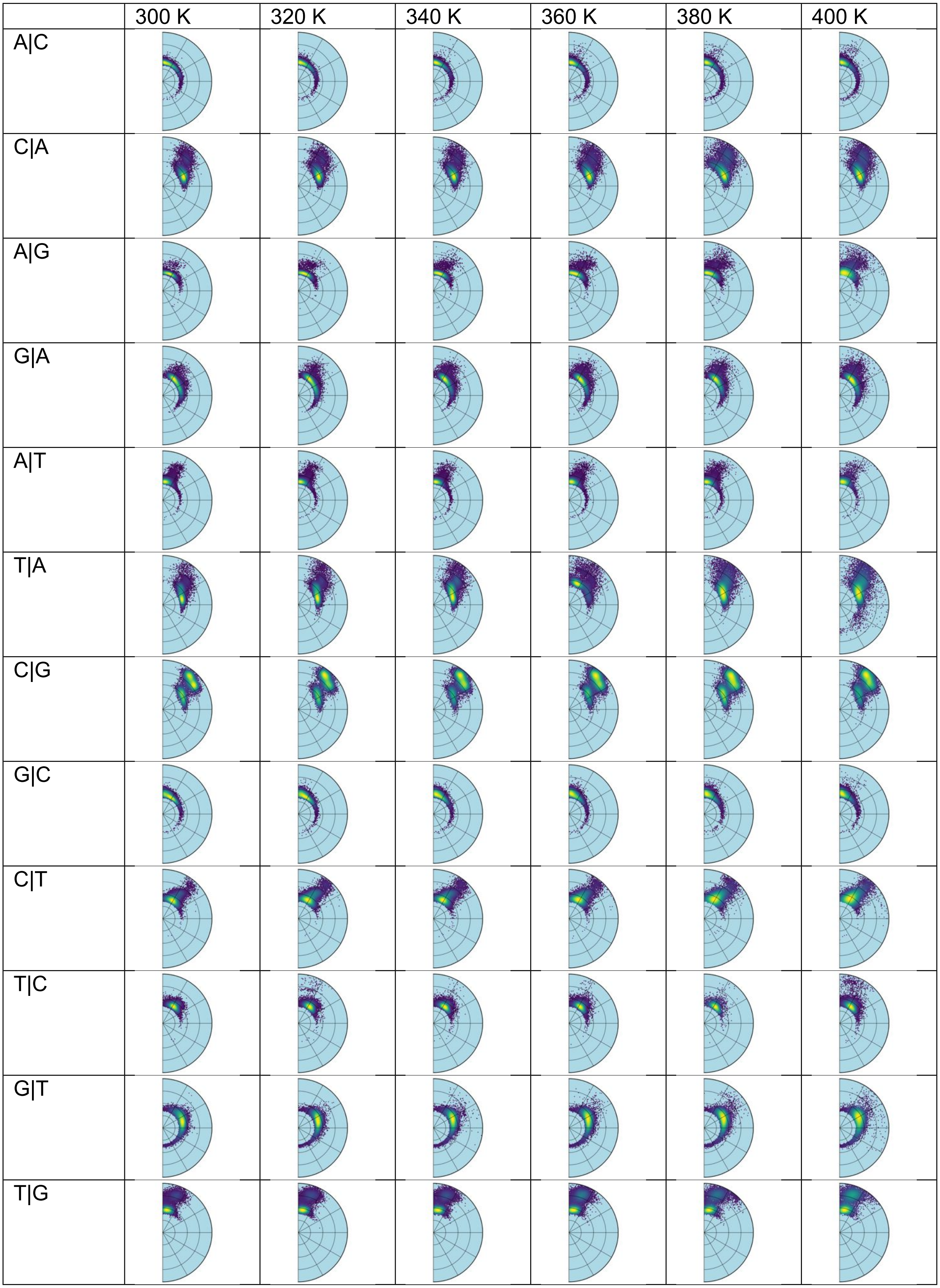

Figure S4: Polar plots for stacked bases in all MD simulations.

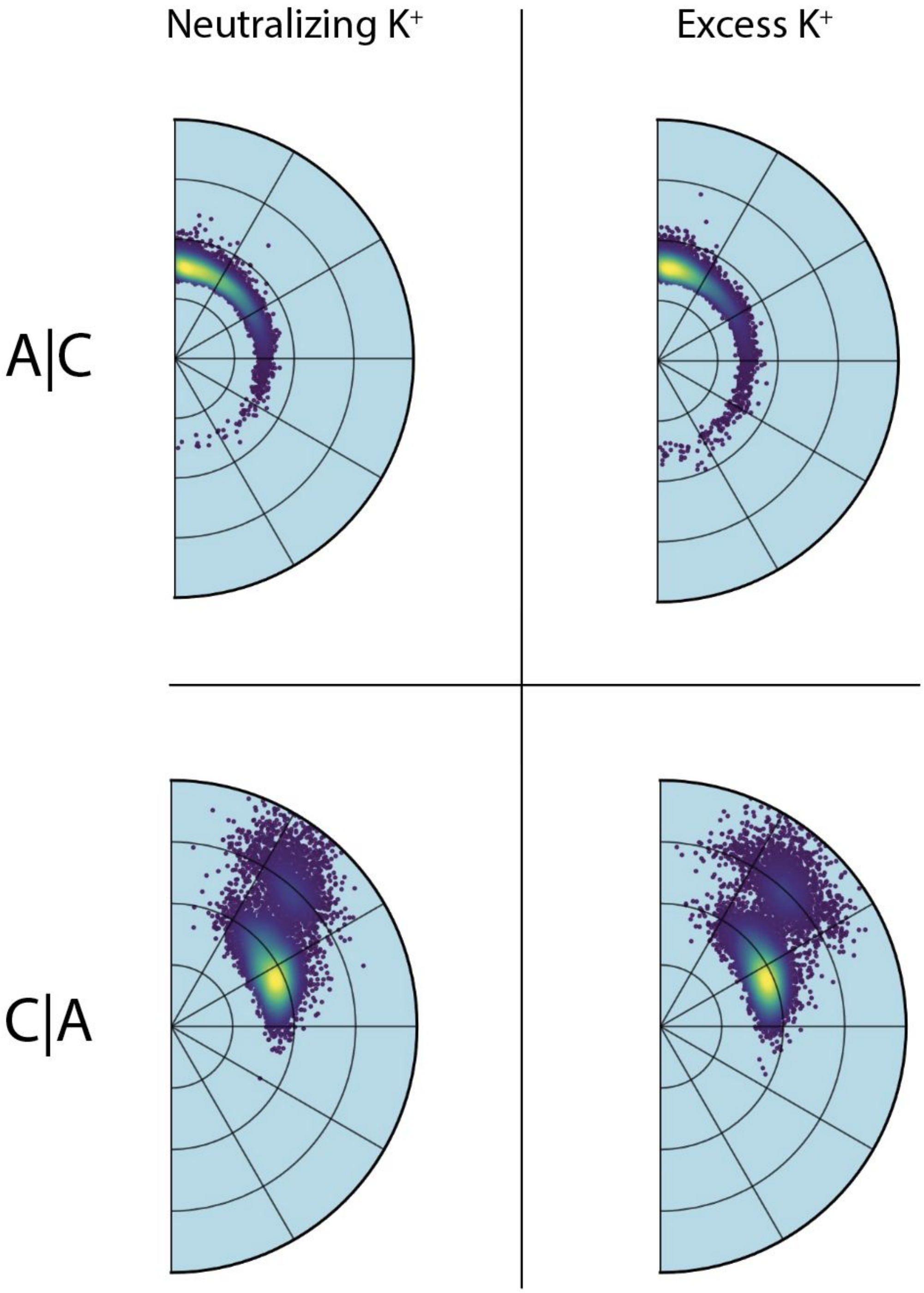

Figure S5: Polar plots for A|C and C|A stacking interactions with K^+ ions at neutralizing or excess concentration.

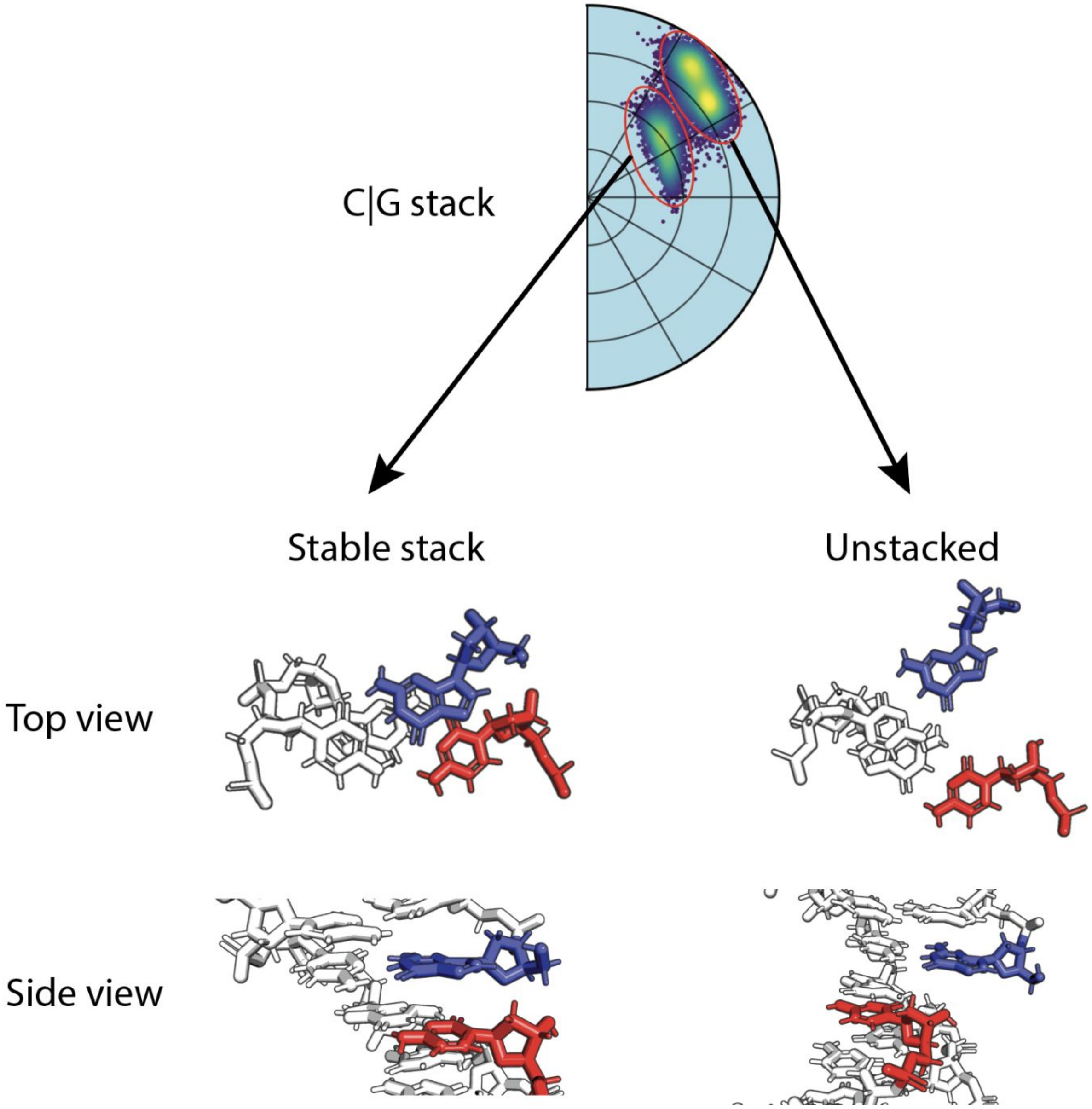

Figure S6: Detailed molecular interface for C|G stacking.
